# The Value of Smartwatches in the Health Care Sector for Monitoring, Nudging, and Predicting: Viewpoint on 25 Years of Research

**DOI:** 10.2196/58936

**Published:** 2024-10-25

**Authors:** Charlotte Köhler, Alexander Bartschke, Daniel Fürstenau, Thorsten Schaaf, Eduardo Salgado-Baez

**Affiliations:** 1 Department for Data Science & Decision Support European University Viadrina Frankfurt (Oder) Germany; 2 Core Unit Digital Medicine & Interoperability Berlin Institute of Health @ Charité Charité - Universitätsmedizin Berlin Berlin Germany; 3 Institute of Medical Informatics Charité - Universitätsmedizin Berlin Berlin Germany; 4 School of Business & Economics Freie Universität Berlin Berlin Germany; 5 Department of Anesthesiology & Intensive Care Medicine Charité - Universitätsmedizin Berlin Berlin Germany

**Keywords:** consumer devices, smartwatches, value-based health care, monitoring, nudging, predicting, mobile phone

## Abstract

We propose a categorization of smartwatch use in the health care sector into 3 key functional domains: monitoring, nudging, and predicting. Monitoring involves using smartwatches within medical treatments to track health data, nudging pertains to individual use for health purposes outside a particular medical setting, and predicting involves using aggregated user data to train machine learning algorithms to predict health outcomes. Each domain offers unique contributions to health care, yet there is a lack of nuanced discussion in existing research. This paper not only provides an overview of recent technological advancements in consumer smartwatches but also explores the 3 domains in detail, culminating in a comprehensive summary that anticipates the future value and impact of smartwatches in health care. By dissecting the interconnected challenges and potentials, this paper aims to enhance the understanding and effective deployment of smartwatches in value-based health care.

## Introduction

The consumer smartwatch market has experienced significant growth during the past years. Initially perceived as lifestyle accessories, smartwatches have transformed into advanced health monitoring devices. Equipped with various sensors, smartwatches capture vital health metrics, such as heart rate, oxygen saturation, fitness levels, and sleep quality. Remarkably, studies suggest that between 76% and 90% of adults are willing to share wearable data with their physician [1-3]. This positions smartwatches as valuable tools for personal health management, providing an unprecedented opportunity for patients to generate and manage their health data actively.

Smartwatches are expected to have a transformative impact on health care, with studies forecasting global cost savings of approximately US $200 billion due to wearable technology [4]. Integrating smartwatch data into electronic health records (EHRs) is a growing trend, enhancing patient monitoring and personalized care. In the United States and Canada, efforts have been focused on incorporating biometric and behavioral patient-generated health data into EHRs [5,6]. These endeavors include developing application programming interfaces (APIs) to address interoperability challenges and to understand social determinants of health better. In Europe, initiatives to integrate smartwatch data also encompass patient portals and shared decision-making tools [5]. A significant development is Germany’s recent legislation, which allows patients to transfer health data collected by smartwatches directly into their health records [7].

Numerous studies explore the integration of smartwatches into health care [8-14]. Yet, many studies concentrate on particular facets or stand-alone applications of smartwatches, often lacking a comprehensive framework that encompasses the diverse challenges and opportunities these devices present. This paper aims to bridge this gap by categorizing and evaluating these challenges within various contexts. By adopting this structured approach, we seek to provide a holistic perspective on the nuances of using smartwatches in health care settings. The goal is to enhance the foundational understanding of these complexities, thereby enabling more strategic and informed decisions regarding the adoption of smartwatches in the pursuit of value-based health care solutions. Furthermore, we hope this framework will guide future studies in clearly identifying the health perspectives they address and in exploring the value and challenges within each specific domain.

We propose categorizing consumer smartwatches into 3 key functional domains: monitoring, nudging, and predicting. Monitoring involves tracking patient health data within medical treatments and providing valuable supplementary insights from data generated outside medical facilities. Nudging refers to individuals using smartwatches for personal health tracking outside of clinical settings, offering significant public health benefits due to self-motivated health tracking. Predicting involves leveraging data from multiple users to train machine learning algorithms for predicting health outcomes, representing a promising area.

Monitoring offers the promise of reaching remote patients and gathering detailed health insights to enhance medical treatment quality. Yet, the effectiveness of this approach hinges on the trust in data-sharing practices and the integrity of the data collected, areas where consumer smartwatches may fall short. In the nudging domain, smartwatches are seen as powerful tools for encouraging healthier behaviors across various demographics, thus playing a significant role in addressing widespread health issues and enhancing public health. However, the responsibility of leveraging smartwatches effectively in nudging scenarios rests on the individual. Predictive use of smartwatches is a nascent yet promising field. Previously, patient data were only collected after diagnosis or during physician visits. Now, smartwatches collect data from both healthy and unhealthy individuals, providing unprecedented insights into disease and health maintenance. However, this area also raises significant data privacy and ethical concerns.

In The Evolution of Smartwatches in Tracking Health section, we will provide a concise overview of the latest advancements in smartwatch technology. Subsequently, we will present the 3 distinct perspectives of smartwatch use, monitoring, nudging, and predicting, exploring each in detail. Finally, we will offer a comprehensive summary, outlining the anticipated value and impact of smartwatches in the health care sector moving forward.

## The Evolution of Smartwatches in Tracking Health

In recent years, the smartwatch market has witnessed a significant surge in sales globally. From generating approximately US $12.67 billion in revenue in 2017, the industry’s earnings soared to US $44.94 billion by 2023, with projections estimating a climb to US $62.46 billion by 2028 [15]. In 2022, Apple led the market as the top-selling manufacturer with their Apple Watch, followed by Garmin’s popular models such as Forerunner and Venu, Fitbit with its Sense smartwatch (as well as together with Google on the Pixel Watch), and Samsung with its Galaxy Watches [15].

All these smartwatches are equipped with various sensors that gather data, which are then analyzed and transformed into health features through each manufacturer’s proprietary algorithms. These sensors often include gyroscopes, magnetometers, barometers or altimeters, GPS, and photoplethysmograms. The study by Henriksen et al [16] provides an exhaustive examination of sensors integrated into smartwatches over time and across different manufacturers. This section will focus on the health features derived from these sensors (passive tracking) while acknowledging that smartwatches also allow users to enter self-reported data (active tracking), such as moods or menstrual cycles, enriching overall health estimation. Promised health features encompass *activity tracking* (eg, daily steps, running, and cycling), *heart rate monitoring* via scanning wrist blood flow, and *electrocardiogram (ECG)*—a newer addition that has received attention for its capacity to detect heart conditions [17] and has thus attracted considerable medical research interest [18,19]. *Fall detection* mechanisms identify abrupt positional changes using accelerometers and gyroscopes. Continuous measurement of blood oxygen and sleep tracking are interconnected features using reflectance photoplethysmography, where optical sensors analyze light rebounding from the wrist [20]. Although sleep tracking has long been available on smartwatches, it is the integration of oxygen level analysis that now enables a truly comprehensive evaluation of sleep quality. This feature is particularly advantageous for managing conditions such as sleep apnea, marking a significant enhancement in sleep health monitoring [21]. The latest sensor addition measures skin *temperature*, supporting passive *cycle and ovulation tracking*—marking a gender-specific health feature innovation.

**Figure 1:**
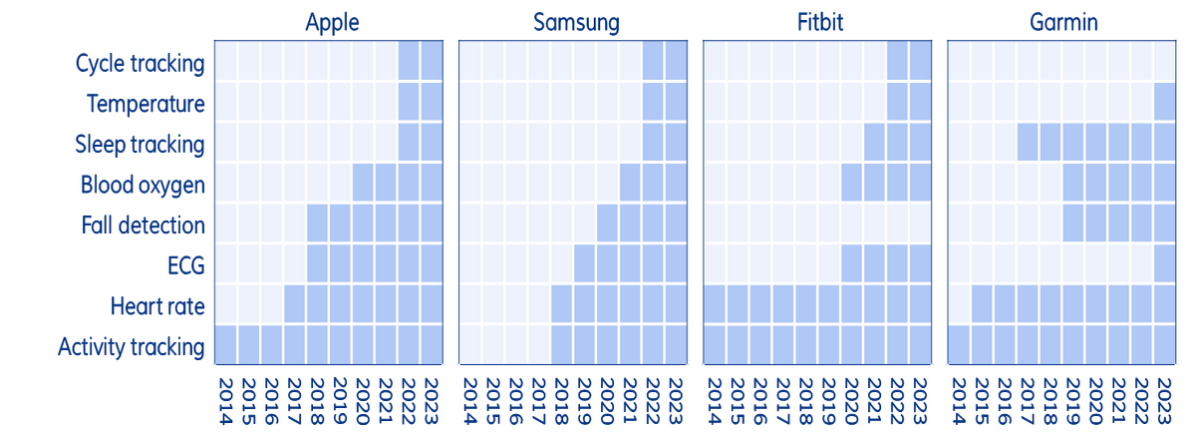
Overview of health tracking features introduced. ECG: electrocardiogram.

Figure 1 illustrates the evolution and adoption of health features across the 4 leading smartwatch brands: Apple, Samsung, Fitbit, and Garmin. A darker shade of blue indicates the year a feature was introduced. Apple stands out as a pioneer, consistently adding new health features and maintaining the most comprehensive offerings. Samsung, initially slower to integrate health features, has rapidly expanded its capabilities since the 2018 launch of the Galaxy Watch, aligning more closely with Apple’s offerings. Fitbit and Garmin, both early adopters of activity tracking, have been slower to incorporate newer health features such as fall detection and ECG. However, they have led in exercise-related innovations, with Fitbit introducing blood oxygen measurement alongside Apple and Garmin excelling in sleep tracking and oxygen level monitoring.

For all the manufacturers mentioned earlier, a smartwatch alone is insufficient for gathering health insights. Users must also connect the watch to a smartphone, install an app, and accept the manufacturers’ data and privacy terms to access detailed health data. This raises concerns about users’ limited control over how their data are handled and used [22]. A detailed study on the privacy issues and potential security threats is provided by Cilliers [23]. While there are numerous health tracking features, it can be assumed that the primary goal of these manufacturers is commercial success, and marketing health improvements serves merely as a means to this end.

As manufacturers continue to enhance their products with more health features, the line between lifestyle accessories and essential health tools becomes increasingly blurred. However, if manufacturers wish to market a tracking feature for medical purposes, they must obtain regulatory approval to classify the function as a medical device [24]. This classification ensures that the features meet the necessary standards for medical use, which is crucial for both consumer safety and product credibility. However, obtaining approval from regulatory bodies such as the Food and Drug Administration in the United States and securing CE marking in Europe is a time-intensive process that can delay the introduction of new features to the market. For example, when Apple introduced the ECG feature, it initially received clearance only in the United States, leaving users in other countries without access at the outset [25]. Consequently, manufacturers try to sidestep these hurdles whenever possible by marketing their functions as fitness and wellness tools rather than for medical purposes, although these features have significant health relevance beyond mere fitness tracking. As of 2024, the development of smartwatches is focused on incorporating several new innovative health features. Functionalities for measuring blood glucose, monitoring blood pressure, and detecting alcohol levels are expected in the upcoming models [26]. These anticipated features highlight an ongoing trend of increasing smartwatches’ relevance in health care.

There is also a growing interest in developing open-source smartwatches, which offer greater control over data privacy and security compared to commercial models and can be tailored for specific health conditions. For instance, Bianchini et al [27] discussed an open-source smartwatch designed for dermatitis management. In addition, smaller manufacturers such as PKvitality and Mindpax [28] have developed smartwatches specifically for continuous blood glucose monitoring and improving treatment outcomes for mental illnesses, respectively.

## Differentiating the Value of Smartwatches in Health Care

### Overview

Critical considerations for using smartwatches in health care include validity (correct measurements) and reliability (consistent measurements); data privacy, ownership, and security; device usability; reimbursement; and the technical integration of data into clinical workflows. Several studies illuminate aspects of these considerations. Validation and reliability studies such as those conducted by Fuller et al [29], Lauterbach et al [30], and Awolusi et al [31] evaluate the accuracy of consumer smartwatches in measuring health metrics. Concerns around data privacy and security are addressed in research by Ching and Singh [32] and Perez and Zeadally [33]. The usability and patient experience with smartwatches are explored in studies by Manini et al [11], Dehghani [34], and Pal et al [35] and the alignment with reimbursement models is explored in the study by Smuck et al [36], while the potential for integrating smartwatch data into treatment is discussed by Dunn et al [37] and Kheirkhahan et al [38]. Notably, the significance of each factor differs depending on its applications in health care. For example, while the precision of step counts may be less critical in nudging scenarios aimed at increasing daily activity, the accuracy of ECG measurements is paramount when used as a determinant for emergency medical care. Our differentiation in smartwatch use—monitoring, nudging, and predicting—provides a framework for evaluating which aspects are more or less crucial depending on the intended health care application.

**Figure 2:**
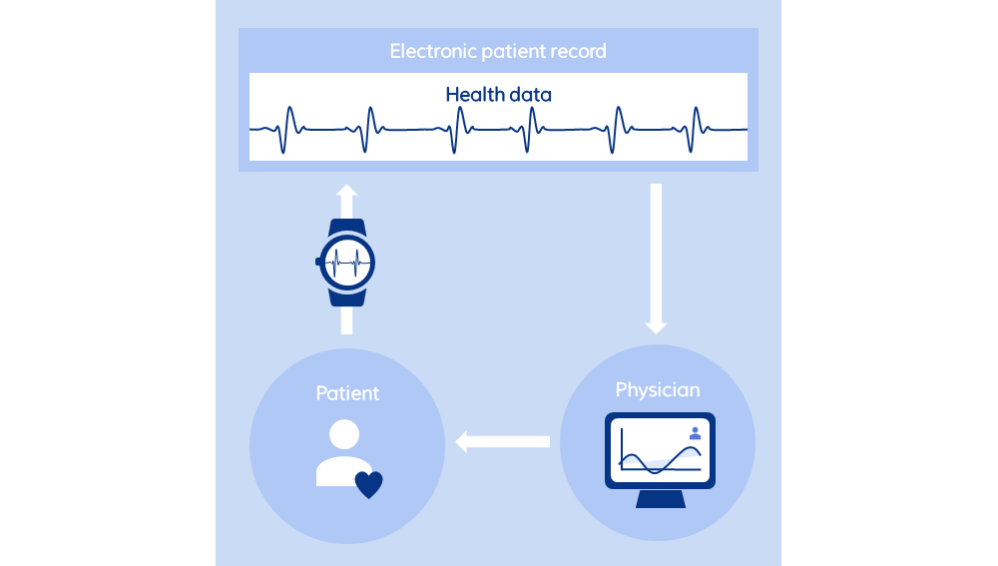
Overview of smartwatch apps in health care (monitoring).

**Figure 3:**
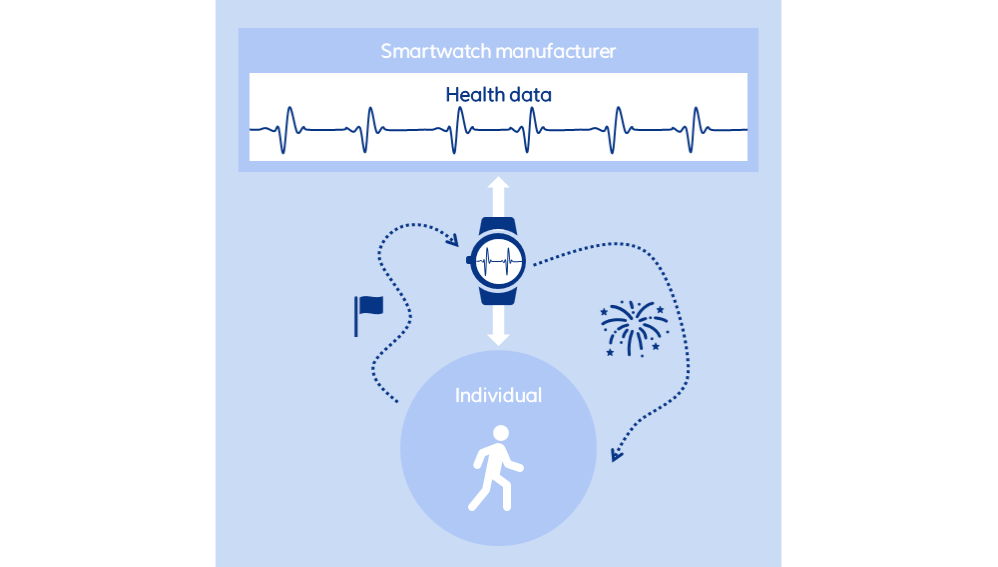
Overview of smartwatch apps in health care (nudging).

**Figure 4:**
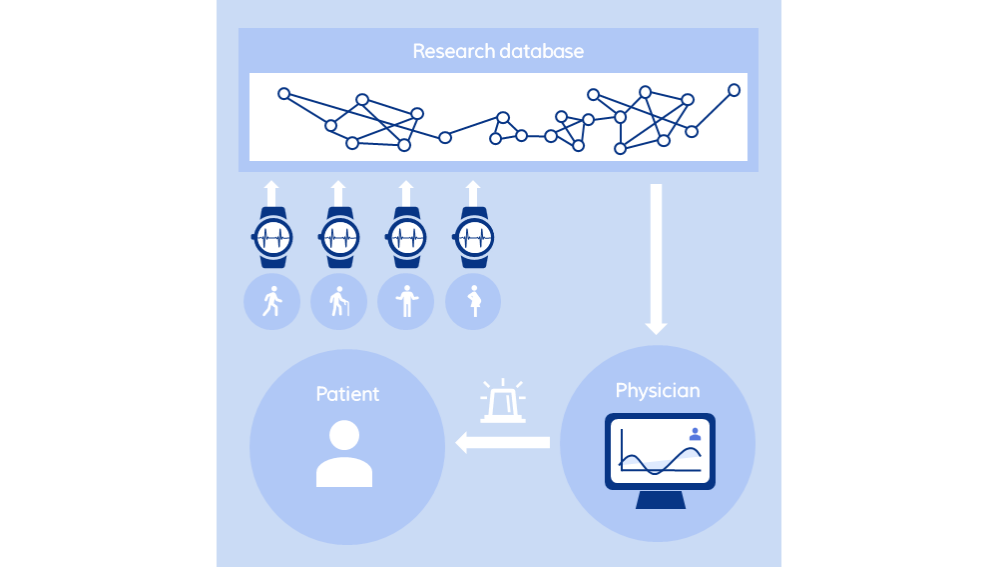
Overview of smartwatch apps in health care (predicting).

In the Monitoring section, we explore the role of smartwatches in patient monitoring, highlighting its benefits and challenges in health care. The Nudging section explores how smartwatches can influence health behaviors through nudging. The predictive capabilities of smartwatches, aimed at predicting health outcomes, are examined in the Predicting section. To illustrate these concepts, Figures 2, 3, and 4 offer visual representations of the three domains. The diagrams highlight the interactions between the individual and the smartwatch, the processes involved in collecting and storing health data, and the healthcare sector’s involvement in leveraging this information.

### Monitoring

#### Overview

Smartwatches provide continuous patient monitoring at low costs and enable the gathering of patient-generated data despite physical separation from medical facilities under normal life conditions, providing health care providers with insights previously unattainable or only accessible through patient self-reporting. For instance, if a physician inquires about a patient’s sleep over recent weeks, traditional answers would rely on subjective estimates. In contrast, a smartwatch can supply objective, unbiased data detailing both the quantity and quality of sleep, as well as its correlation with other vitals measured concomitantly. This significant advancement in the precision and reliability of health assessments is undeniable and represents a transformative shift in the delivery of care. In addition, the ability of smartwatches to automatically transmit vital sign assessment and movement to the EHR remotely can free up staff from manually collecting vital signs, allowing physicians and nurses to spend more time with hands-on care and avoiding patients’ unnecessary visits to health institutions.

To implement effective monitoring of a patient’s vital data, the process begins with establishing and discussing targeted health outcomes with the patient, ensuring alignment with their ongoing therapy. The technical capabilities of potential smartwatches can be discussed; if the patient already possess a smartwatch, its suitability for monitoring specific biometrics is checked. Subsequently, the appropriate sensor data from the smartwatch are selected to match these outcomes, and a precise data collection interval is set. Data are synchronized with a medical database, for instance, stored within the patient’s EHR. Health care providers are then granted access to a dashboard offering in-depth data analyses, thus providing critical insights into the patient’s health status. Regular follow-up consultations enable the health care team to monitor progress toward the established goals. Any deviations from the expected outcomes are explored jointly with the patient to adjust the treatment plan as needed. This cyclical and collaborative process champions a patient-centric approach, ensuring that the monitoring initiative is customized to suit the individual’s unique health requirements and circumstances effectively (Figure 2).

In the subsequent paragraphs, we will initially explore the significance of smartwatches within the monitoring domain, both as a stand-alone and in comparison to other domains. This will be followed by examining the challenges and obstacles linked to using smartwatches for monitoring purposes.

#### Supervised Smartwatch Use to Provide Access to Health Data That Were Previously Unavailable, Costly, or Difficult to Obtain

Using smartwatches as monitoring tools is predominantly advantageous in 3 scenarios. First, smartwatches introduce the capability to gather health data that were previously either unavailable, costly, or difficult to obtain. Traditionally, continuous health monitoring has been confined to hospital stays or medical practices. Given the high costs, minimizing in-patient duration is a priority. Accessing patient parameters before or after hospital admission could significantly enhance care quality by making previously uncollected data available to physicians. An example can be found in the study by Randazzo et al [39], where patients were equipped with a custom-developed smartwatch that is designed to collect essential vital signs, including ECG, heart rate, blood oxygen levels, and temperature. The primary goal is to facilitate remote monitoring by hospitals, enabling earlier discharge of patients while still ensuring close observation of their biometrics. Aside from hospital stays, other monitoring methods, such as visiting a sleep laboratory for sleep apnea detection, can be inconvenient and burdensome for patients. In their study, Chen et al [40] demonstrated that a Huawei smartwatch can distinguish between normal sleep patterns and sleep apnea events. Although the study acknowledged the smartwatch’s limitation in providing more detailed classifications, it emphasized that even this initial level of differentiation can contribute to reducing the number of hospitalizations required. Fall detection for the older adults is among the most studied monitoring applications of smartwatches (refer to studies by Mauldin et al [41], Casilari and Oviedo-Jiménez [42], and Brew et al [43]). The severity of falls, particularly those occurring at night or when individuals are alone, is significantly increased due to the individuals’ inability to seek help. This often results in prolonged periods of distress, as they may have to endure pain for an extended time before the incident is noticed. In addition to monitoring and fall detection, smartwatches can also assist in fall risk assessment and prevention [44] (refer to the Predicting section).

Second, smartwatches are revolutionizing health data accessibility, particularly for underserved populations and areas where traditional monitoring is challenging due to geography, economy, or limited digital health literacy. Research by Sheng et al [45] and Ibeneme et al [46] underscores these disparities. Enhanced by mobile software, smartwatches are crafting new pathways for care, reaching beyond typical consumer bases. For instance, the Apple Watch is favored by families with older members for its emergency services and health data–sharing capabilities, as noted by Tai et al [47] and Strauss et al [48]. Similarly, it is proving beneficial for children with conditions such as cardiovascular diseases or autism, offering precise monitoring that rivals traditional methods [49-51]. Furthermore, smartwatches are essential in settings such as extreme environments, remote workspaces, and professional sports, delivering critical insights into users’ physiological states and safety [52-54]. This leap forward in monitoring technology is reshaping health care delivery, particularly for remote or rural communities, highlighting an urgent need for expanded research into how telemedicine and wearable technology can synergize to enhance care for all.

Third, monitoring solutions can be particularly beneficial when enhanced data on a patient’s status could either improve care quality or alleviate the illness’s burden. This is particularly crucial for individuals managing chronic conditions that significantly impact daily life. Chronic diseases such as diabetes or mental health issues exemplify areas where smartwatch monitoring can make a meaningful difference. Chakrabarti et al [55] highlighted the utility of smartwatches for patients with diabetes by monitoring symptoms such as frequent urination and sleep disturbances, which are challenging side effects of the condition. In mental health care [56], there is growing interest in using smartwatches to predict and manage psychotic disorder relapses and to monitor behavioral health. The use of smartwatches in the monitoring domain has a prevalent advantage compared to the other domains discussed later, which is the supervision of a health care professional when patients interact and use the smartwatch. Spender et al [57] claim that there are several reliability issues of the data collected with smartwatches that can be connected to wrong wearing behavior or choice of smartwatch, such as the specific device used, any changes to the device during the monitoring period (such as updates), the manner of device use, and how the device is worn. When a patient is under the supervision of a physician, potential errors in measurement and use can be more effectively monitored, and patients can be better educated on the proper use of the smartwatch. This supervision ensures that data collected for health monitoring are more accurate and reliable, mitigating issues related to device inconsistencies or incorrect use. Smartwatches reduce treatment costs across various applications; even in cases where the integration of these tools increases costs, the improvements in patient quality still deem these tools overall effective from a value-based health care perspective [58].

The digital capabilities of smartwatches enable health care providers to extend their reach to more patients, particularly in remote areas or specific patient groups. Consequently, this leads to fewer in-person visits and more effective use of health care resources. Leveraging smartwatches to monitor patients with chronic illnesses presents a cost-effective solution, potentially mitigating high treatment expenses. However, economic evaluations must be conducted carefully, tailored to each setting, and compared against other telemedicine alternatives. Nevertheless, the level of patient data detail gathered by these tools is unmatched. In addition, the introduction of smartwatches as monitoring tools in health care can significantly enhance the delivery of patient-centered care. These devices have the potential to empower patients to actively engage in decision-making processes about their health in accordance with their values and preferences. This increases their agency in managing different aspects of their health and illness, helping to reduce fear, and facilitating the involvement of family and friends when needed. Smartwatches, being a low-cost tool and often subsidized by health insurance companies, can contribute to providing more equitable access to personal health management allowing health care professionals to observe, prevent, or respond to health issues based on data generated in everyday life outside medical facilities, enhancing access to care and reducing patient vulnerability.

#### Smartwatch Use in Treatment: Trust in the Treatment Process, Data Sharing, and Data Quality

The quality of vital parameters collected is crucial for effective health monitoring. Generally, smartwatches receive positive validation for their measurement accuracy. For instance, recent studies such as the study conducted by Zarak et al [59] report that ECG measurement accuracy in consumer smartwatches ranges from 65% to 99% and the study by Jiang et al [60] indicates that the accuracy of saturation of peripheral oxygen (SpO_2_) measurements varies between 90% and 96%. However, the real-world applicability of these devices in health settings is questionable, as most validation tests are conducted in laboratory settings [29]. For example, step counts can become inaccurate for patients using gait aids [61], and there is diminished performance in smartwatches for individuals with darker skin tones [62]. This may lead to patients’ reluctance to trust these devices and physicians’ reservations about recommending smartwatch use.

Another crucial aspect of fostering trust in new technology is considering the data privacy of collected health data. Patients must be informed that smartwatches gather highly personal health information, which, despite being treated with the utmost sensitivity, poses privacy concerns. Therefore, it is essential for patients, in collaboration with their physicians, to conduct a thorough cost-benefit analysis when considering the use of smartwatches for continuous health monitoring. This process is akin to evaluating standard treatments, such as medications, where the potential side effects are balanced against the anticipated health benefits in consultation with health care providers. The issue of data privacy does not belong to a specific medical domain; however, it creates uncertainty over who bears the responsibility of educating patients on this vital matter. This underscores the need for clear guidelines and educational efforts to ensure patients can make informed decisions about using smartwatches in their health care regimen [63].

### Nudging

#### Overview

With nudging, we refer to the process where individuals analyze their own data to actively improve their health. Nudging involves subtly guiding individuals toward behavior change, with the aim of fostering long-term habit modifications that result in improved health outcomes [64]. It entails using psychological strategies rather than direct medical interventions to influence user behavior as shown in the study by Marchiori et al [65]. Smartwatches play a significant role in this process by tracking fitness parameters and providing motivational cues, enhancing individuals’ physical and psychological self-awareness. This heightened awareness can lead to greater recognition of health-promoting behaviors, such as improved nutrition habits, even if the smartwatch’s sensor technology may not directly measure these behaviors [66]. Unlike in the monitoring settings, no direct supervision or contextual integration in the health care setting is typically associated with nudging using smartwatches. However, incentives from health care providers, such as health insurers or physicians, may exist to encourage the adoption of smartwatches (Figure 3).

Smartwatch providers strategically incorporate health-related benefits and outcomes into their marketing efforts and design decisions. For instance, the smartwatch interface may include visual rewards to incentivize users to achieve activity goals, adhere to sleep and nutritional schedules, and engage in mindfulness exercises. Sangameswaran [67] showed that smartwatches, being worn close to the body and serving as a personal device, offer effortless access to information and use subtle methods, such as haptics, vibrations, and light, to capture the wearer’s attention. These features make smartwatches a powerful tool for driving behavior changes throughout the day. For example, the Apple Watch uses a “firework” visualization as a reward when users successfully reach their daily goals for steps, exercise, and standing. Similarly, Garmin watches provide users with information about their stress levels, emphasizing the significance of mental awareness and well-being. Smartwatches can guide users through breathing exercises, enabling them to incorporate meditation practices into their daily lives. An overview of related digital nudges can be found in the study by Bergram et al [68].

In the following sections, we consider the benefits and challenges in the nudging domain.

#### Effect of Smartwatches on Healthy Behaviors Across a Broad Demographic

Smartwatches, with their low barrier to entry, are well-positioned to impact a broad demographic, particularly in public health domains. They hold significant potential in managing lifestyle-related diseases—commonly associated with high stress, inadequate sleep, and insufficient physical activity that are prevalent across diverse populations.

First, smartwatches serve as educational tools promoting a healthier lifestyle. While a trusted physician-patient interaction is invaluable for lifestyle guidance, smartwatches offer similar, continuous, and motivational support. Conditions such as hypertension and obesity, widespread in Western societies, can be alleviated through increased physical activity, an area where smartwatches have shown efficacy. Research indicates that smartwatch ownership often leads to a significant uptick in physical activity. For instance, Ferguson et al [69] documented an increase of up to 40 minutes in daily walking time, while Li et al [70] emphasized smartwatches’ positive effect on user activity levels. Smartwatches facilitate personal goal setting and progress tracking, engaging users in their health journey. This self-driven approach empowers individuals to take charge of their health, fostering a sense of achievement and personal responsibility.

Second, smartwatches provide nudging for individuals who lack external motivation, such as the older adults, who may experience loneliness and thus have diminished social incentives to remain active. In situations without external encouragement, smartwatches adopt a supportive role, offering controlled encouragement. Studies such as the study by Chung et al [71] show that older participants feel motivated by their smartwatches to be more active, illustrating the devices’ capability to fulfill a motivational void. Boateng et al [72] developed a smartwatch step-counting app specifically tailored for the older adults to engage them in physical activity. In addition, the study by Rosales et al [73] revealed the intrinsic motivation among the older adults to use smartwatches, associating the device with feeling “cool” and youthful.

Similar to the monitoring domain, significant potential cost savings are anticipated in both the monitoring and nudging scenarios. However, accurately quantifying the financial benefits of health nudging via smartwatches presents challenges, mainly due to the long-term nature of these effects and their interaction with other health initiatives. Although measuring the direct health outcomes of nudging can be complex, the broader impact on public health is anticipated to be considerable. This perspective is supported by actions from major German health insurers who offer reimbursements for smartwatches, typically cautious in supporting preventive measures, thereby acknowledging their utility in health interventions. On average, these insurers provide a subsidy of approximately 300 Euros (US $328) toward the purchase of smartwatches, demonstrating a recognition of their value in enhancing health care outcomes and saving health-related costs [74].

#### Distribution of Health Benefits of Smartwatches and the Onus of Using a Smartwatch for Healthy Behavior

In addressing the challenges of smartwatch use for health-related nudging, maintaining user engagement is paramount. The role of usability, which includes technical quality, design, and affordability, is critical in this regard. As Chuah et al [75] suggest, user motivation to achieve personal goals greatly influences continued smartwatch use. However, there is a mismatch between the demographics of typical smartwatch users and those who would benefit most from their health applications. For instance, the user group with the highest use is between 25 to 34 years, a demographic at lower risk of lifestyle-related diseases. In contrast, for older individuals, who could greatly benefit from such technology, smartwatch use is considerably lower. This use pattern highlights the need for targeted strategies to increase smartwatch adoption among older populations, despite disparities in digital literacy, who stand to gain significantly in terms of health monitoring and management [76]. Similarly, the study by Helsen et al [77] highlights a pronounced connection between runners and smartwatch use. This implies that individuals already engaged in healthy activities such as running are more inclined to use smartwatches. Therefore, these devices may be predominantly adopted by those who have already committed to a health-conscious lifestyle. While owning a smartwatch can facilitate increased activity levels, its effectiveness hinges on the individual’s preexisting motivation to adopt life-changing health behaviors. Constantiou et al [78] highlight the individualized use and impact of smartwatches on health tracking. Users’ reactions to their performance data can vary greatly; unsatisfactory feedback might lead to discouragement and reduced use, whereas those receiving positive feedback are likely to find smartwatches particularly beneficial, reinforcing their already positive health behaviors. This distinction underscores the personalized nature of smartwatch utility and its varied effects on individual health tracking and motivation efforts.

However, not only the equitable promotion, design, and adoption among different user groups but also the technical fairness of smartwatches raise important considerations. It has been observed that the performance of smartwatch sensors can vary significantly across various skin tones, and their accuracy tends to decrease in individuals with higher BMI [79]. This variability in sensor performance not only raises questions about the inclusivity and fairness of these devices but also about their effectiveness and reliability in health monitoring across diverse populations. This issue underscores the need for more inclusive design and testing processes in the development of smartwatch technology to ensure that it serves a broad demographic, regardless of their physical characteristics.

Another factor that should not be overlooked is that smartwatches, though expected and discussed to bring major health improvements, can also be harmful and decline the health of people. This is particularly discussed for mental health, that is, patients experiencing anxiety, for which the permanent monitoring of biometrics can have a negative effect [80]. In the context of nudging, where users interact solely with the smartwatch without direct supervision from health care providers, there exists a risk associated with the use, interpretation, and implementation of smartwatch recommendations. These risks could potentially lead to harmful behaviors or increase expectations, pressure, or stress. Without professional guidance, users might misinterpret health data or recommendations, leading to decisions that could adversely affect their health rather than improve it.

Another significant challenge is the requirement for users to accept privacy and data processing terms set by manufacturers, often after the purchase. This situation can leave users susceptible, having no option but to agree to these terms to use the device. It places the onus of data management and interpretation on the users themselves, without a collective regulatory body overseeing manufacturer practices in health applications. The widespread use of smartwatches for health purposes thus imposes a responsibility on providers to guard against manipulative or harmful nudging practices [81]. This situation calls for stringent measures in data ownership, privacy, and transparency about the nudging mechanisms used, particularly because manufacturers’ primary focus is on commercial success rather than patient health.

### Predicting

#### Overview

In the domain of prediction, we focus on detecting potential health risks and predicting future health outcomes for individual patients [82]. We identify 2 main directions that strengthen the use of predictive methods with smartwatch data. First, the ease of continuous data collection from patients enriches the understanding of their health statuses. Using these data can improve predictions of treatment outcomes, drawing from patient’s own historical data or that of others with similar conditions. This approach is closely aligned with the monitoring domain, as it leverages patient-monitored data to predict risks and outcomes. Second, health data collection has traditionally been concentrated around visits to health care providers, primarily capturing data from those actively receiving treatment. This method leaves a significant gap as healthy individuals who do not need to visit physicians remain underrepresented in health datasets. Relying exclusively on data from individuals with a disease can result in biased insights; including data from healthy individuals provides a more balanced view, helping to understand why certain people remain healthy while others develop illnesses.

The outlined procedure in the prediction domain starts by consolidating data from individuals, collected with the smartwatches, into a medical research database. As an initial step, the desired outcome must be defined; this could be a classification task (eg, “cancer” vs “healthy” as in the study by Hou et al [83]) or a regression task (like calculating a risk score as in the study by Goldstein et al [84]). Given that smartwatches collect time-series data, the outcome of interest could also be a change in the observations over a period or a time output itself (eg, predicting relapses as in the study by Moshe et al [85]). Once the outcome has been formulated, the appropriate prediction model must be selected. In this context, supervised machine learning, a major subset of artificial intelligence (AI) algorithms, is used extensively for making predictions. These algorithms are trained on one segment of the data and tested on another, ensuring they can effectively identify patterns and make accurate predictions based on new, unseen data. However, there is often a trade-off between model performance and explainability. For example, decision trees provide a clear set of decision rules, which makes the decision-making process understandable and increases the trust in results; however, they typically only perform good prediction results for simpler datasets. In contrast, deep learning algorithms, which use billions of decision nodes, often lack transparency in explaining outcomes but can yield superior outcomes even for complex prediction scenarios.

When developing a predictive model, evaluation metrics are crucial for determining which model to apply. One important metric is accuracy, which indicates the overall correctness of predictions. In medical predictions, however, there is often a higher interest in not just the general prediction accuracy but also the accuracy of specific predicted classes and potential mistakes made by the model. For example, consequences of incorrect predictions vary significantly (eg, mistakenly telling a patient they are healthy when they need treatment is generally worse than the reverse). Therefore, sensitivity and specificity, precision, and recall, which measure how well positive and negative classes are predicted, are commonly considered as well.

Insights derived from these analyses are then reviewed by health care professionals or embedded in clinical decision support systems (CDSSs), facilitating improved patient health outcomes (Figure 4). The study by Steyerberg and Vergouwe [86] outlines the requirements for developing prediction models in a clinical environment. Many of these steps align with established data science principles, and it is particularly noteworthy that the authors evaluate specific metrics, such as the model’s suitability and usefulness for a particular CDSS.

We will explore the most advantageous scenarios for using smartwatches in predictive applications, followed by an examination of the related challenges.

#### Using Predictive Methods for Major Advances in Health, Particularly in Preventive Care and Risk Estimation

Predictive analytics can significantly enhance the quality of care for patients in treatment management by using smartwatch data for informed decision-making. For instance, Stojancic et al [87] used smartwatch data to predict pain scores in patients with sickle cell disease, thereby facilitating more effective pain management. Similarly, Liu et al [88] applied predictive analytics to anticipate mortality events in palliative care using smartwatch data, easing the emotional burden for patients and their families by providing some certainty in a challenging situation. In addition, smartwatches are gaining recognition for managing mental health conditions, particularly those with high-relapse risks such as schizophrenia, as explored in the study by Fonseka and Woo [89]. These examples illustrate that for patients already undergoing treatment and monitored via smartwatch, the application of predictive algorithms can further enhance the quality of care. In addition, Brasil et al [90] highlighted that applying AI to analyze large datasets can address key challenges in rare disease management with limited patient populations and geographical dispersion. Brasil et al [91] provided a comprehensive review of machine learning algorithms tailored to rare disease studies.

The most intriguing and still uncertain aspect of predictive analytics is its potential to predict conditions in individuals not yet undergoing treatment, thereby playing a significant preventive role. This potential unfolds in 2 key ways: first, by diagnosing diseases that are already present but undetected as well as identifying risks for diseases not yet manifest; and second, by analyzing data from individuals with a disease at an aggregated level to optimize health care resource assignment and capacity management.

For the first time, predictions allowing for a timely diagnosis could make treatment possible before an irreversible stage is reached. This might be particularly relevant for diseases that are asymptomatic in the early stages [92]. Many examples can be found for cardiovascular diseases. In their systematic review, Nazarian et al [93] showed that based on smartwatch data cardiac arrhythmias can be predicted with an average accuracy of 97%. A comprehensive review of both custom-built and commercial smartwatches for detecting cardiovascular disease risk is detailed in the study by Moshawrab et al [94], which highlights a rapid increase in research over the past 3 years, pointing to a promising future for deeper insights in this area. Furthermore, Mizuno et al [95] emphasize the importance of using these devices for cardiovascular disease risk prediction. The authors suggest that in cases of heightened risk, continuous monitoring combined with lifestyle changes, akin to the nudging scenario presented earlier, can be particularly effective. An overview of additional diagnostic applications of wearables, including in psychological and neurological diseases, liver diseases, metabolic diseases, and sleep disorders, is provided in the study by Chakrabarti et al [55]. The most notable example of using smartwatch data at an aggregated level for resource management occurred during the COVID-19 pandemic. For instance, Föll et al [96] demonstrated how smartwatches could be used as risk-scoring tools in general wards for patients with COVID-19, optimizing resource allocation to those most in need. Similarly, Esmaeilpour et al [97] developed an alert system using smartwatches to detect respiratory viral infections and manage available capacities more effectively. Similar ideas can be found in the studies by Zhu et al [98], Mishra et al [99], Kallel et al [100], and Abbasi [101]. A notable initiative in Germany [102] solicited smartwatch data from the public, leveraging it to predict and identify COVID-19 outbreaks nationwide without direct testing or patient contact. This project highlighted the smartwatch’s significant role not only in detecting outbreaks but also as a strategic tool for policy makers in pandemic management. Despite its success, the project concluded in 2022, and a new platform for data donation has not yet been established. To effectively harness the value of these data and implement predictive measures in health care, regulatory approval and both public and private reimbursement are essential, not only for COVID-19 but also for future public health crises [103].

#### The Value of Predictive Smartwatch Use and Its Implementation Into Feasible Solutions

After having discussed how leveraging smartwatch data for predictive analytics can aid not only in treating diseases but also in preventing them. However, to date, we do not see feasible large-scale implementation of collecting and using smartwatch data for predictions in health care.

A major challenge in using smartwatch data for predictive analytics is the privacy and ethics of data collection. We have already highlighted the need for patients to balance the benefits of sharing their smartwatch data against potential risks in the Monitoring section. In addition, in this section, we have discussed the importance of collecting data not only from patients but also from healthy individuals. For the latter, the direct benefits of sharing their personal data are not always clear, raising questions about whether the risks of data sharing are justified. Informed user consent in sharing their personal data for predictive purposes is critical, highlighting a significant responsibility and a gap in effectively educating the public about data sharing [63]. Both digital literacy and, more specifically, data literacy [104], are crucial for understanding the implications of data sharing. Gupta et al [105] observed that older and more conservative participants were less inclined to share their data, whereas Seifert and Vandelanotte [106] found that those comfortable with technology and higher incomes were more willing. Moreover, Gupta et al [105] emphasized that the willingness to share data extends beyond simply signing a consent form; factors such as data deletion policies, regulatory oversight, and transparency are also crucial for individuals to agree to share their data. With rapid advancements in AI, it becomes challenging for individuals to agree to data sharing without knowing the potential future uses of their data [63]. Data privacy measures necessitate a focus on data minimization and anonymization. However, it is essential to recognize that excessive anonymization can strip away valuable contextual information vital for making accurate predictions, as demonstrated by Hicks et al [107], who showed that activity and health patterns can vary significantly across different demographics.

Given these substantial challenges, data donation models could provide a viable solution by collecting data only from individuals who voluntarily choose to participate, significantly increasing the likelihood that they understand the implications of their decision. Gomez Ortega et al [104] proposed a framework for establishing a data donation platform, detailing strategies for setting up the platform and engaging users to continuously evaluate and reconsider their participation, thereby enabling ethical data sharing. The initiative undertaken by the Robert Koch Institute during the COVID-19 pandemic is a prime example, where smartwatch data donations were solicited to track potential outbreaks, demonstrating this method’s ability to reach a broader demographic than traditional research methods [102]. However, Strotbaum et al [108] argued that the successful establishment of such a platform raises long-term issues that have yet to be resolved. These include data anonymization, primary and secondary uses of data, benefits for donors, and access to these datasets—challenges that persist from the perspectives of both researchers and policy makers. Solutions and regulations for these issues need to be developed in the future.

## Discussion

In exploring smartwatches’ impacts on health, we dissected their roles across monitoring, nudging, and prediction domains. This section consolidates those insights, providing a summarizing lens to juxtapose and compare challenges across these areas. We specifically focus on the predominant challenges highlighted in the Differentiating the Value of Smartwatches in Health Care section: the validity and reliability of measurements, data privacy and security, device usability, and technical integration.

Challenges related to the *validity and reliability* of data from smartwatches are significant, as these devices rely on algorithms that are often nontransparent and proprietary to their manufacturers. Conducting empirical studies to assess the accuracy of devices from leading brands could help provide some reassurance on using these in a medical setting [29,59,60]. However, due to the fast-paced release cycle of new models, by the time studies such as the study by Fuller et al [29] are published, the evaluated models are often no longer produced or available to consumers. As this strategy essentially follows a “test afterward” approach, a more proactive alternative could involve exerting stricter regulations on manufacturers to disclose their algorithms or establishing uniform testing standards that all manufacturers must meet before releasing new models. We also see potential in specifically engineered smartwatches for medical applications by smaller manufacturers. These devices usually adhere to more stringent regulations and offer greater transparency about their algorithms. However, they cannot compete with the range of functions offered by major brands such as Apple, Garmin, and Samsung. Particularly in the domains of nudging and predicting, where strong motivation to wear the device is necessary, we see no alternative but to rely on these established major smartwatch providers.

*Data privacy and security* pose substantial challenges in the context of sensitive health data collection by smartwatches. The burden of data protection often falls on patients, without a straightforward way to circumvent the storage of their data on manufacturers’ servers. In both monitoring and prediction scenarios, where consumer smartwatches are used, the data are typically replicated to health care providers or research databases. Nonetheless, users are required to consent to the sharing of their data with manufacturers. Within the nudging domain, the data exclusively reside with the manufacturers, introducing significant risks. As outlined in the previous section, the value of data collected by smartwatches is immense. While the health care system is subject to stringent regulations for data storage, manufacturers may store data indefinitely, potentially including sensitive information, such as the frequency of falls, mental health issues, or heart attacks. While major manufacturers such as Apple, Samsung, and Garmin currently guarantee a safe environment for personal health data [109-111], past incidents of data breaches [112] show that there is always a risk. Furthermore, users may share their data with third-party apps on their smartphones that do not implement the same level of security measures, distributing highly sensitive health data to many providers and increasing the risk of a breach at some point.

This scenario underscores the need for rigorous data protection measures and scrutiny over how health data collected by smartwatches is stored, used, and shared in commercial domains. However, with increasing medical relevance, manufacturers might be incentivized to provide alternatives. For instance, Garmin is exploring the use of localized data transmission hubs that store data solely on the user’s device and allow direct transfer only into the EHR, thereby reducing the risk of unauthorized access to the data.

The *usability* of smartwatches impacts both the quantity and quality of data collected. According to the study by Ding et al [113], enhancing usability in medical settings could be achieved through simpler interfaces and extended battery life. While smartwatches designed as lifestyle accessories are advantageous, those crafted specifically as medical devices often lack the design and motivational features of mainstream smartwatches. Usability becomes crucial when motivation is necessary, or health benefits are not direct and long-term and hence not immediately apparent. Therefore, in nudging and predicting scenarios, encouraging users to wear their smartwatches consistently is vital. In contrast, in monitoring scenarios, where wearing a smartwatch is directly connected to personal health improvements, users may be more inclined to tolerate less user-friendly devices, provided they deliver the anticipated health outcomes. In considering the usability of smartwatch technology, it is crucial to highlight the issue of *access*. It is worth remembering that consumer smartwatches aim to maximize sales within their target demographic, yet this group does not always align with those who could most benefit from the health improvements offered by the technology. There is a notable use bias toward White individuals, adults aged 18 to 50 years, and those with higher education levels [114]. Despite the potential of smartwatches as a low-cost, accessible health care solution, their actual use across different demographics is uneven. Financial barriers are one obstacle which health care providers can address to some extent. However, educational efforts are also essential to integrate smartwatches into a broader range of individuals’ health routines, ensuring more equitable access and utilization.

*Integration* stands out as a pivotal element in smartwatch applications across monitoring, nudging, and predicting. It is crucial to differentiate between device connectivity and data interoperability. For connectivity, smartwatches use various protocols such as Wi-Fi, Bluetooth, NFC, ZigBee, ANT+, Cellular, and USB to transfer data, chosen based on data volume, transmission frequency, and impact on battery life. Internet-enabled devices, often smartphones, serve as gateways for data transmission to caregivers, facilitating Cloud connections and communication. However, most smartwatches are compatible with a few specific platforms, such as Apple’s watchOS, Google’s Wear OS, and other proprietary systems [115]. Cross-platform interoperability faces hurdles; for instance, while Wear OS devices can pair with iOS, functionality is reduced, and features such as ECG or blood pressure monitoring may be unavailable. Apple’s watchOS, designed exclusively for iOS integration, further limits system openness and compatibility with other devices and platforms. Health data exchange is tightly regulated, necessitating compliance with international standards for integration into EHR. Achieving semantic interoperability involves adopting universal terminologies such as Systematized Nomenclature of Medicine Clinical Terms (SNOMED CT) and Logical Observation Identifiers Names and Codes (LOINC), while syntactic interoperability requires standards such as Health Level 7 Fast Healthcare Interoperability Resources. Although there has been progress in health data standardization, aligning smartwatch data with these protocols remains a challenge, even with efforts such as the standards developed by the Institute of Electrical and Electronics Engineers or the guidelines provided by the Food and Drug Administration [116].

While our discussion has explored the different areas of smartwatch applications separately, we have yet to encounter a solution that satisfactorily addresses all 3 areas. Ideally, a single smartwatch that meets all these challenges and requirements would significantly enhance overall health and blur the lines between these categories. Until such a solution is available, it is crucial that users and patients are thoroughly informed about the benefits and risks associated with using wearables for health data tracking. This is particularly pertinent in the nudging domain, where individuals independently attempt to interpret health data using a device on their wrist. Nudging domain, a gap in health care knowledge exists, which future developments must consider. Bridging this gap is essential to ensure that individuals have the necessary understanding and tools to effectively use smartwatches in managing their health.

### Conclusions

In this paper, we have explored the multifaceted value of consumer smartwatches within the health care sector, categorizing their utility into monitoring, nudging, and predicting domains and highlighting their potential in transforming health and illness management. Monitoring leverages smartwatches for gathering patients’ data, including vital signs and activity data. Nudging pertains to the capability of smartwatches to influence users toward adopting healthier lifestyles, such as increasing physical activity, which can significantly enhance their long-term health. Predicting extends these applications by using vast amounts of data collected from numerous users to train AI models and implement CDSSs based on them. For each domain, we have outlined the associated benefits and challenges, presenting a comprehensive analysis of these elements.

While numerous challenges persist in integrating smartwatches into health care, our findings indicate that these challenges vary in intensity and risk across the different domains of monitoring, nudging, and predicting. Overall, we would like to emphasize the following aspects.

First, in the literature, smartwatches are often discussed in general terms regarding their potential and challenges, which can create the impression that the obstacles to their use are insurmountable. However, our contribution aims to demonstrate that challenges significantly vary across different domains. A more nuanced understanding of domain-specific challenges can help stakeholders realize that not all issues are equally relevant, thereby facilitating the targeted implementation of smartwatches. We advocate for a clear distinction in the future use of smartwatches within medical settings, specifying the particular domain of application as we have suggested. This approach will ensure that the use of smartwatches is appropriately tailored to the relevant medical context. By adopting this perspective, smartwatches should not be viewed as a universal tool. While they may be versatile in certain cases, it is crucial to first consider the specific scenarios in which they will be integrated and used to maximize their effectiveness and relevance in the delivery of health care.

Second, we have demonstrated the immense potential value of smartwatches for health across various domains. However, despite numerous studies highlighting this value, these solutions are rarely implemented in individuals’ daily lives. To achieve safe and beneficial integration into health care settings, it is crucial to develop practical strategies. This involves creating methods for individual-level integration, such as personalized health management apps that use smartwatch data, improving interoperability with existing health care systems, and training health care providers on the effective use of smartwatch data. In addition, exploring the broader potential of collecting extensive health data from the population with minimal effort, as demonstrated during the COVID-19 pandemic, could significantly enhance public health monitoring and response.

Third, data privacy is a critical concern when it comes to the use of smartwatches. While these devices can collect a variety of health metrics and serve as diagnostic tools, most consumer smartwatches lack the standards required for securely handling these private data. As a result, the health data collected by smartwatches are one of the most unprotected sources of health information, despite its detailed and sensitive nature. Currently, the responsibility for protecting these data is largely placed on individuals who use smartwatches. To address this issue, there is an urgent need for standardized data protection protocols, stronger encryption methods, and greater involvement from organizations and regulatory bodies to ensure robust safeguards are in place, thereby relieving individuals of this burden.

While smartwatches have been a part of the consumer landscape for years, their integration into health care is still new. Following the study by Heidel and Hagist [117], their long-term effects must first be demonstrated throughout the next years. Future economic studies will be crucial for assessing smartwatches from a value-based perspective, aiming to quantify their demonstrated impact on enhancing care quality and determining the costs required to realize this value. The technological landscape of smartwatches is rapidly advancing. At present, various innovative features are under development for future market introduction. Significantly, the progression of sensors tailored for health data collection stands out, with upcoming functionalities including blood glucose and blood pressure monitoring. This ongoing evolution undoubtedly establishes smartwatches as crucial components of health care delivery now and moving forward.

## Abbreviations

AI: artificial intelligence
API: application programming interface
CDSS: clinical decision support system
ECG: electrocardiogram
EHR: electronic health record
LOINC: Logical Observation Identifiers Names and Codes
SNOMED CT: Systematized Nomenclature of Medicine Clinical Terms
SpO_2_: saturation of peripheral oxygen
